# Pilot SERS Monitoring Study of Two Natural Hypersaline Lake Waters from a Balneary Resort during Winter-Months Period

**DOI:** 10.3390/bios14010019

**Published:** 2023-12-29

**Authors:** Csilla Molnár, Teodora Diana Drigla, Lucian Barbu-Tudoran, Ilirjana Bajama, Victor Curean, Simona Cîntă Pînzaru

**Affiliations:** 1 National Institute for Research and Development of Isotopic and Molecular Technologies, 67-103 Donath, 400293 Cluj-Napoca, Romania; 2 Biomolecular Physics Department, Babeş-Bolyai University, Kogălniceanu 1, 400084 Cluj Napoca, Romania; teodoradrigla@yahoo.com (T.D.D.); ilirjana.bajama@ubbcluj.ro (I.B.); 3 Electron Microscopy Centre, Babeș-Bolyai University, Clinicilor 5-7, 400006 Cluj-Napoca, Romania; lucian.barbu@itim-cj.ro; 4 Faculty of Pharmacy, “Iuliu Hatieganu” University of Medicine and Pharmacy, Victor Babes 8, 400347 Cluj-Napoca, Romania; curean.victor@elearn.umfcluj.ro; 5 Institute for Research, Development and Innovation in Applied Natural Sciences, Babes-Bolyai University, Fantanele 30, 400327 Cluj-Napoca, Romania

**Keywords:** Raman, SERS, salt lakes monitoring, balneary resorts, in situ, PCA

## Abstract

Water samples from two naturally hypersaline lakes, renowned for their balneotherapeutic properties, were investigated through a pilot SERS monitoring program. Nanotechnology-based techniques were employed to periodically measure the ultra-sensitive SERS molecular characteristics of the raw water-bearing microbial community and the inorganic content. Employing the Pearson correlation coefficient revealed a robust linear relationship between electrical conductivity and pH and Raman and SERS spectral data of water samples, highlighting the interplay complexity of Raman/SERS signals and physicochemical parameters within each lake. The SERS data obtained from raw waters with AgNPs exhibited a dominant, reproducible SERS feature resembling adsorbed β-carotene at submicromole concentration, which could be related to the cyanobacteria-AgNPs interface and supported by TEM analyses. Notably, spurious SERS sampling cases showed molecular traces attributed to additional metabolites, suggesting multiplexed SERS signatures. The conducted PCA demonstrated observable differences in the β-carotene SERS band intensities between the two lakes, signifying potential variations in picoplankton abundance and composition or environmental influences. Moreover, the study examined variations in the SERS intensity ratio I_245_/I_1512_, related to the balance between inorganic (Cl^−^-induced AgNPs aggregation) and organic (cyanobacteria population) balance, in correlation with the electrical conductivity. These findings signify the potential of SERS data for monitoring variations in microorganism concentration, clearly dependent on ion concentration and nutrient dynamics in raw, hypersaline water bodies.

## 1. Introduction

For centuries, hypersaline water bodies worldwide have been revered for their therapeutic benefits, yet comprehensive information on water composition, dynamics, and correlations with healing properties remains limited [1,2,3].

**Figure 1:**
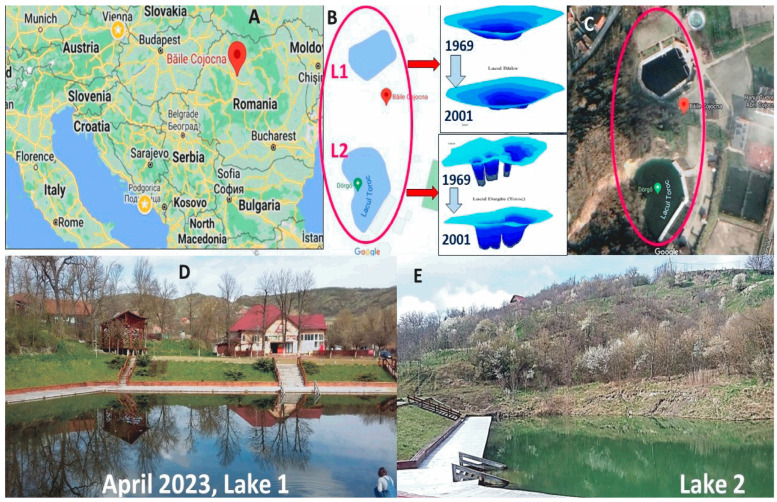
Geographic location of salt lakes L1 and L2 from Cojocna Băi resort, Romania (**A**–**C**), as snapped from Google Maps, and photographs of the lakes taken in April 2023, as indicated (**D**,**E**). Insertions in B are adapted from Serban et al., 2005 [5], and show the dynamic reduction of lake depth between 1969 and 2001, with about 1 m in 10 years.

Cojocna Balneary Resort in Cluj County, Transylvania, Romania, comprising two hypersaline lakes, the Bathing Lake, also known as Török Lake (L1), and the Great Lake (L2, Figure 1), is an example of a natural hypersaline water resource and an excellent study environment. The lakes are located on former salt mines that have collapsed and filled with water, resulting in the dissolution of salt from halite bedrock by underground springs in salt mine pits. Currently, the bathing lakes are popular touristic and balneotherapeutic destinations in the metropolitan proximity area of Cluj-Napoca, Romania, not only in summertime but also during the cold season, due to the indoor heated saltwater pool, supplied with water from the L2, along with the newly settled infrastructure for balneotherapeutic procedures. 

The Cojocna lakes comprise a cluster of interconnected bodies of water, whose bathymetric and morphometric characteristics were investigated in the early 1970s for the first time by Romanian geographer Teodor Pinzaru and published in 1971 in the local Studia Geographia Journal of Babes-Bolyai University from Romania. The publication [4] is only available as a print copy in the university library and highlights that Lakes 1 and 2 were partly set up for bathing and spa-therapeutic treatment. Later, Serban et al. [5] showed that the salt lakes from Cojocna revealed very active dynamics, and the reduction of the lake depth is about 1 m in 10 years (see insertion in Figure 1), and the salinity differs from one lake to another.

The region’s geological history attributes Cojocna to a Miocene-age marine salt deposit [6,7], giving rise to unique hypersaline ecosystems with distinct properties [6,8,9]. Several physicochemical properties associated with microbial diversity and mud analysis of these lakes have been investigated [7,8,9,10,11,12], and different opinions about salinity, electrical conductivity, pH, and microbial communities are found in these point studies. Despite their reputation, the therapeutic properties of Cojocna’s salt lakes remain largely undocumented, primarily due to a lack of comprehensive information on the chemical composition and dynamics of these waters. Secondly, there is a notable absence of substantial medical evidence demonstrating the curative effects on patients. While standard physicochemical and biological parameters are available for regulatory purposes in the indoor facility of the resort, there is a notable absence of basic information on outdoor water bodies for the public.

The previously reported salinity levels showed variability, ranging from 31.4 ppt for L1 and 31.2 ppt for L2 [9] to the other reported value of 101.01 g/L [10]. The high salinity of these lakes creates a favorable environment for hosting halophilic microorganisms represented by archaea, bacteria, and eukarya that require high salinity conditions to survive, hence their name “salt-loving.” They are commonly found in hypersaline waters and require sodium ions for their growth and metabolism, typically in salt concentrations greater than 3%, equivalent to the average ocean level, which has about 3.5% NaCl [13]. Alexe et al. [8] reported that the surface waters of hypersaline lakes, with salinity levels exceeding 10%, are dominated by halophilic communities. 

The majority of halophilic microorganisms, including bacteria and archaea, produce photosynthetic pigments, which, in dense populations of halophiles, generate the pink, red, or purple color of water bodies [14]. However, Cojocna lakes never showed the coloration associated with algal blooming, their visual appearance ranging from dark, opaque blue-green to dark green or even black. The abundance and diversity of microorganisms in these lakes are remarkable, with Lake 1 being observed to always be greener and darker than Lake 2, suggesting a higher concentration of photosynthetic microorganisms, although both lakes benefit from similar climatic conditions. These visual and empiric observations triggered questions about their potentially distinct health benefits. Thus, the frequent public question, “Which lake is better for bathing for my health condition?” remains unanswered due to the lack of scientific information. 

The present study employing Raman and SERS study of lake waters refers to the winter months only, to exclude the anthropogenic influence through touristic and balneary exploitation during the warm months, from May to October.

This paper introduces an innovative approach, using normal Raman spectroscopy with 532 nm laser line excitation to measure the raw salt water spectra from the first-meter surface layer, which is the only one exposed to bathing cure. Further, SERS analyses of waters are conducted, aiming to prospect for sensitive detection of organic compounds and to investigate any correlation between Raman and SERS data. Usually, in salty environmental waters, normal Raman spectra exhibit a typical sulfate band [12] at 979 cm^−1^ assigned to the sulfate stretching mode [12] among the water bands. Other molecular anions are rarely detectable due to their low concentration and high fluorescence background in water. But excitation with the 532 nm line may produce resonance Raman spectra of carotenoids from photosynthetic microorganisms, thus reflecting the microorganism population. Investigating correlations between these spectroscopic Raman and SERS data sets might reveal additional insight into the inorganic/organic balance of waters without laborious extraction and separation procedures, which require high chemical consumption. Raman and SERS data are analyzed in conjunction with the pH and conductivity measurements during the monitoring period to address the monthly composition and dynamics of surface waters.

Many studies have demonstrated the usefulness of Raman spectroscopy for the identification and characterization of pigments and biomarkers produced by microorganisms [15,16,17,18,19,20,21,22], although debates regarding unambiguous species detection remain due to their Raman complex signature dependencies on many environmental factors as well as the experimental conditions. An interesting recent pilot study employing confocal Raman microscopy in situ reported by Wieser et al. (2023) [22] highlighted the importance of chemometric tools to be able to accurately characterize the biological dynamics of algae material on different culture days for algae exploitation, demonstrating significant differences along the algal growing time and detecting chemical changes in cells without extraction and separation needed. Such an approach would also be suitable for monitoring the algal-rich environmental water bodies on a larger scale. The drawback is still the implementation of such an automatic approach that requires a robust chemometric component to become user-accessible. 

The SERS technique has so far been less employed to study environmental water bodies [23]. Although apparently abundant literature linking “SERS + environmental waters” is available, addressing a wide range of pollutants, pharmaceuticals, dyes, toxins [24,25,26], or other harmful substances spiked in waters and subsequently detected and eventually quantified for limited concentration ranges, or addressing novel nanostructured substrates with increased enhancement factors, SERS is yet blamed for a lack of interlaboratory operability, reproducibility, and standardization procedures that are not yet recognized as an agreed technique for monitoring programs. Obviously, a lack of data sets regarding SERS in environmental waters does not allow for realistic conclusions on SERS operability for such monitoring purposes. 

In this paper, we focus on the SERS of salt lake waters, with multiple goals: (i) to assess the capability of Raman spectroscopy to provide real-time and in situ monitoring information directly beneficial for the ongoing monitoring of the salt lakes, to capture changes in the composition and dynamics of the lake waters as they occur; (ii) to link raw Raman data of waters with their corresponding SERS signature; (iii) to investigate potential correlations between classical physicochemical parameters and Raman/SERS output of waters; and (iv) to draw conclusions on the relevance of such a monitoring approach for transferability to stakeholders related to balneary treatment in salt waters. These objectives faced scant knowledge of the salt lake properties, just to mention that the only salinity data in the existing literature was contradictory [9,10]. 

To the best of our knowledge, this is the first SERS pilot monitoring study for environmental water bodies.

## 2. Material and Methods

### 2.1. Silver Colloid Synthesis and Characterization

Materials required for the preparation of the colloid, the silver nitrate, and the sodium citrate were purchased from Sigma-Aldrich. As the SERS active surface, a sodium citrate silver colloid prepared according to the standard procedure reported by Lee and Meisel [27] was employed. Briefly, 45 mg of silver nitrate was dissolved in 150 mL of triply distilled water and brought to a boil. A solution of 1% sodium citrate (5 mL) and 100 mL of pure water were added to the boiling solution and allowed to continue boiling for an hour. The resulting AgNPs stock has been freshly characterized using UV-VIS electronic spectroscopy and electron microscopy techniques (SEM and TEM), and their usual features in terms of absorbance, morphology, and size distribution have been in full agreement (Supplementary Information, Figures S1 and S2) with previously reported and used similar AgNPs stocks [23,24,25,26,28]. The Raman spectrum of blank colloid showed only a weak band of water, as expected. AgNP stocks were freshly prepared at the beginning of the study and carefully preserved in dark and cold conditions for SERS monthly monitoring experiments on lake waters. The SERS sampling protocol followed a similar approach by adding 10 µL of raw lake water sample to 500 µL of colloidal AgNPs and measuring them immediately within three replicated independent samples from each water batch. 

### 2.2. Sample Collection and Preparation

Raw water samples from the two adjacent salt lakes have been collected monthly in triplicate from each lake, one sample from 1 m depth and two samples from surface waters (about 15 cm depth) during the cold season from November 2022 to April 2023, resulting in 36 raw water samples (2 lakes × 3 samples per lake × 6 months). The bathing water level only (first meter depth) was taken into account since this layer is the only one accessible for balneary bathing due to its high salinity. 

Water pH, temperature, and electrical conductivity were measured in situ at the time of collection. Lake water was sampled in a 500 mL vial (three per lake) and immediately transported to the laboratory and stored in cold and dark conditions, while subsamples were prepared for Raman and SERS immediate measurements (same day). We obtained written permission for experimental sampling through a collaboration contract signed with the authorities responsible for the management of the Balneary Lakes at Cojocna Resort.

The micro-Raman spectra were recorded from liquid water samples by drop coating deposition Raman (DCDR). Droplets of 10 µL from each water sample were placed on a hydrophobic, Teflon-coated stainless steel µ-RIM^TM^ slide from BioTools. 

The SERS samples were made by adding 10 µL of raw water samples to 500 µL of silver colloidal solution and measured in glass vials of 2 mL with a cap. The SERS samples were measured in triplicate with a portable instrument.

The samples collected in April were used for TEM measurements. For TEM measurements of the same SERS samples, we used probes prepared in a similar way to the SERS samples, from which 7 µL suspension droplets were deposited onto 300 mesh carbon-coated copper grids.

### 2.3. Instrumentation

The electrical conductivity and pH of the samples were measured on site with HQ40d equipment (HACH USA, multi-analyzer). The conductivity resolution measuring range is 0.01 µS/cm - 0.1 mS/cm and the pH resolution is 0.001/0.01/0.1 pH.

A Renishaw InVia Reflex confocal Raman system has been employed for micro-Raman spectra acquisition. A Cobolt diode-pumped solid-state (DPSS) air-cooled laser operating at 532 nm has been employed for Raman excitation (100 mW) via a Leica research-grade microscope with a 5× objective. Detection was achieved with a RenCam CCD detector with 1024 × 256 pixels and a spectral resolution of 0.5 cm^−1^.

The optical absorbance of the obtained silver nanoparticles and collected water samples was measured using a UV-Vis Shimadzu 1900 spectrophotometer.

The SERS spectra were recorded using a rapid Wasatch Photonics fully modular Raman Spectrometer (WP 532-A-SR-IC) with a 532 nm line for excitation and an output power of 10 mW. SERS spectra were recorded in triplicate in the spectral range 100–3200 cm^−1^ with a spectral resolution of 14 cm^−1^. The experiments were conducted three times using different samples prepared from the same batch. All the measurements have been performed in triplicate at room temperature.

Transmission electron microscopy in conjunction with energy dispersive spectroscopy (TEM-EDX) was done on a Hitachi SU8230, Japan, cold field emission scanning transmission electron microscope.

### 2.4. Data Processing and Statistical Analysis

Data processing has been achieved using OriginPro 2021b, OriginLab Corporation, Northampton, MA, USA. Averaged raw Raman spectra were calculated from three independent subsamples of each case and further used for analytical purposes. For surface waters, the averaged spectral data of the two diametral sampling locations on each lake was further used for the statistical analysis. To this averaged Raman spectrum, another one has been added, referring to the "depth" of the water (1 m). Thus, 2 lakes × 2 samples each × 6 months resulted in a total of 24 sample data sets for statistics. To calculate the Pearson correlation coefficient from the Raman datasheet, the band intensity at 981 cm^−1^ attributable to sulfate stretching mode has been used, while for SERS spectra, the 245 cm^−1^ strong and omnipresent signal attributable to Ag-Cl (due to the chloride ions-induced aggregation of AgNPs) has been considered, both being relevant for the “inorganic” spectral signature of waters. The strong SERS signal at 1512 cm^−1^ attributable to β-carotene C=C stretching mode was used as being relevant for the organic, microorganism-generated spectral counterpart. These spectral band intensity values were analyzed regarding any correlation within Raman-SERS as well as each Raman technique with the sample pH and electrical conductivity values. These parameter values were measured three times for each lake at the water collection point and further used as averaged values for each date of collection. 

The statistical analysis, including correlation analysis and principal components analysis (PCA), was conducted in the R programming language version 4.2.1. For this purpose, the package ggcorrplot library version 0.1.4.1 was used to visualize Pearson’s correlation coefficients among various physio-chemical properties (electrical conductivity and pH) and Raman and SERS signals of six-month-collected water samples [29]. PCA was performed using the FactoMineR package version 2.8 [29,30]. Results from the PCA were finally visualized using the factoextra library version 1.0.7 [31]. The PCA analyses for differentiation of the two distinct lakes were conducted on a dataset comprising the calculated SERS relative intensity values of the characteristic band at 245 cm^−1^ and the β-carotene C=C stretching mode at 1512 cm^−1^. The PCA analyses conducted to discriminate physicochemical properties with SERS involved the intensity ratio I_245_/I_1512_ and the measured electrical conductivity and pH values of the water samples. 

For all statistical analyses, the raw datasets, without normalization and background subtraction procedures, were used. For comparative SESR data presentation, spectra were background subtracted and normalized to units. 

## 3. Results

### 3.1. Physical and Chemical Characteristics

**Figure 2:**
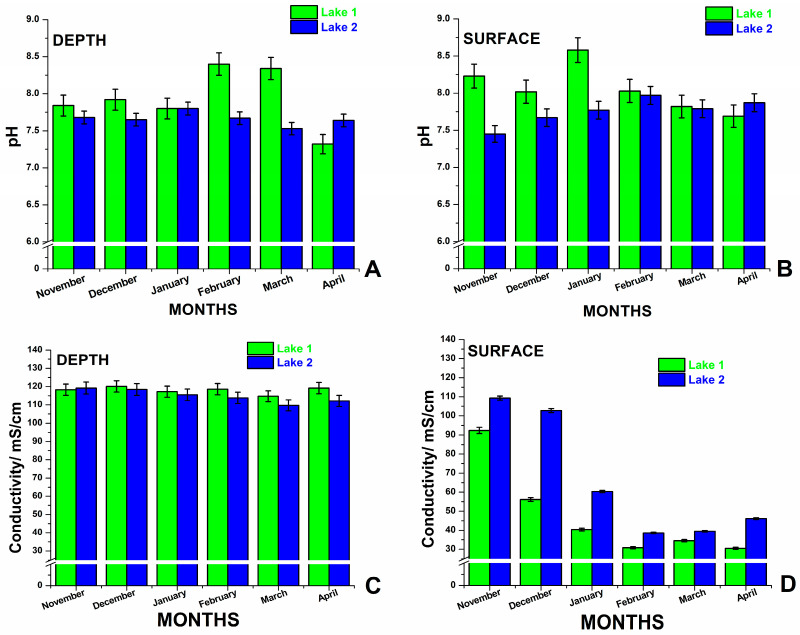
The in situ measured pH and electrical conductivity of salt waters from Lake 1 and Lake 2 at 1-meter depth (**A**,**C**) and at surface (**B**,**D**) during winter months from November 2022 to April 2023. Error bars represent a percentage of data values. The color legend always corresponds to green for Lake 1 and blue for Lake 2, as indicated on each graph. Error bars represents 1.94 % of value.

The measured physicochemical parameters, including temperature, pH, and electrical conductivity, were monitored in situ, and their monthly variability over the study period has been summarized in Figure 2.

The water temperature in the lakes varies depending on the season, ranging from around 4.7 °C to 23.2 °C (Figure S3) during the winter months at a depth of 1 meter, and a substantial increase in temperature was observed in April.

The pH levels of the lakes ranged between 7.2 and 8.5 (±0.1) (Figure 2A,B). Specifically, the pH values in Lake 1, with the exception of the month of April, were consistently higher than those in Lake 2, both at the surface and at the depth layer. For Lake 2, the pH values did not surpass 8, fluctuating between 7.2 and 8 throughout the studied period. In contrast, the pH values for Lake 1 ranged between 7.3 and 8.6 (±0.1) over the six-month duration.

The level of electrical conductivity in water (Figure 2C,D) can indicate the presence of dissolved substances, chemicals, and minerals (for example, potassium, magnesium, and sodium, carbonate, chloride, and sulfate). Higher amounts of impurities lead to higher electrical conductivity. Even a small quantity of dissolved salts and chemicals can raise the electrical conductivity of water [28].

The lakes have a high salinity level, with varying electrical conductivity depending on the season and depth. The electrical conductivity fluctuates a lot seasonally in both lake surface waters, but in a 1-meter-deep area, only a slight variation can be noticed. At the depth level, the electrical conductivity is higher each month than in the surface waters, which means a higher salt level. Surface water conductivity exhibited a wide variation, ranging from 30 to 110 µS/cm, with the lowest values observed between January and April. Conversely, at a depth of 1 meter, the conductivity level ranged between 100 and 120 µS/cm. Notably, in Lake 1, the conductivity was found to be lower than that of Lake 2 at the surface layers, whereas the inverse was observed at the deeper level.

In the context of water analysis, UV-VIS spectroscopy was employed to detect and quantify the carotenoid and chlorophyll content. These exhibit characteristic absorption spectra in the UV-Vis region. Carotenoids absorb light in the visible range, particularly in the blue and green regions, resulting in their distinctive color. Chlorophyll, on the other hand, absorbs strongly in the blue and red regions of the spectrum, with minimal absorption in the green region, which gives algae their green appearance [32].

The absorbance of water samples from two lakes was measured from the UV-Vis spectra using incident light of a wavelength equal to 470 nanometers for carotene and 668 nanometers for chlorophyll, according to the literature [33]. The results are shown in Table S1 and Figure S4.

### 3.2. Raman Spectra of Raw Waters from the Two Lakes of Cojocna Balneary Resort during Winter Months from November 2022 to April 2023

**Figure 3:**
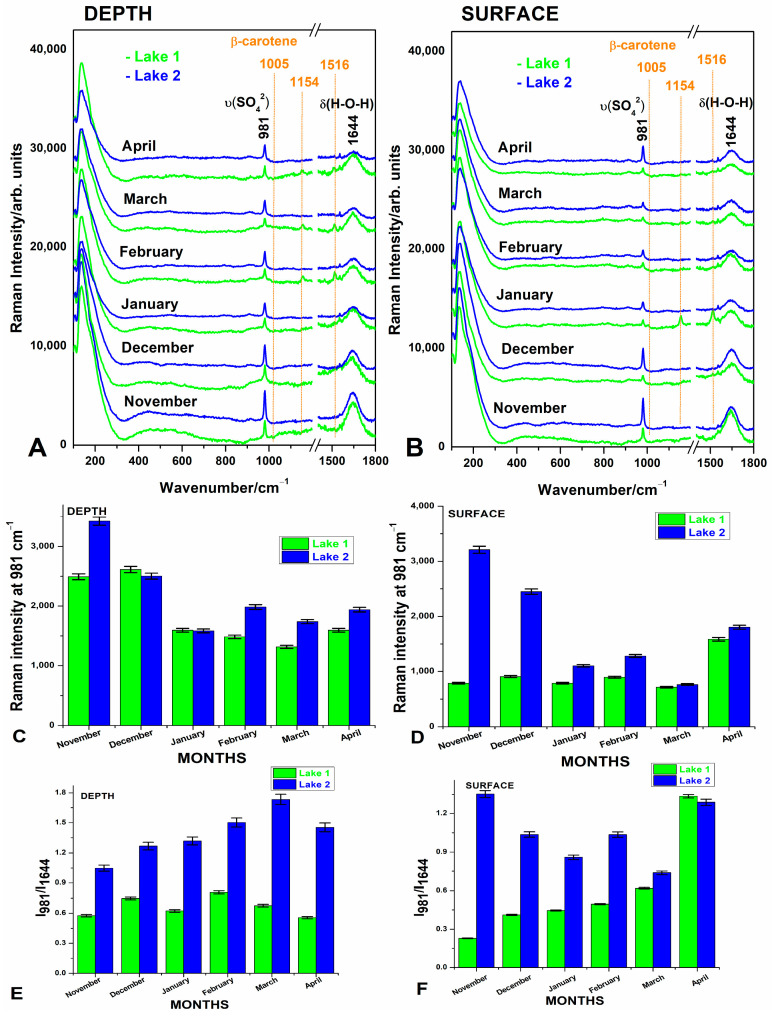
Raman spectra of the Lake 1 and Lake 2 waters from the 1 m depth (**A**) and the surface waters (**B**) sampled over the six-month winter period, from November 2022 to April 2023. The plots in (**C**,**D**) represent the comparative display of the intensity of the Raman band of sulfate at 981 cm^−1^ for Lake 1 (green) and Lake 2 (blue) in samples from 1 m depth (**C**) and in surface waters (**D**) for each month, as indicated. The ratios calculated from the intensities at 981 cm^−1^ band and water O-H band at 1644 cm^−1^ in the Raman spectrum show the variation of sulfate concentration relative to water band and reflect the monthly salinity balance of salt lakes in depth (**E**) and in surface waters (**F**). Error bars represent the percentage of data values. Vertical offset in (**A**,**B**) was applied for clarity. The positions of the carotenoid bands are highlighted with vertical orange lines. They occur in several “green” spectra, referring to Lake 1 waters. The Raman spectra exhibit prominent bands at 981 cm^−1^, which have been attributed to the symmetric stretching mode of sulfate [12,23], along the typical Raman band of water at approximately 1644 cm^−1^ (δ(H-O-H). Comparative display always shows higher intensity band of sulfate in the “blue” spectra, referring to Lake 2, in all cases. The relative intensity of sulfate to water bands (**E**,**F**) is predominantly > 1 for Lake 2, while for Lake 1, it is always <1. The variation of the sulfate band intensities at 981 cm^−1^ is shown in (**C**,**D**) according to the months, from 1 m depth and surface, respectively. Thus, the normal Raman spectra clearly distinguished the inorganic content differences between lakes over the monitoring period. Monthly comparison between “green” and “blue” spectra showed different features. The Raman spectra of raw water from L1 “green” showed the additional weak bands of carotenoids at 1005, 1154, and 1516 cm^−1^ [23,24] in many cases, suggesting higher photosynthetic microbial abundance in Lake 1.

Figure 3 presents the Raman spectra of waters from the two lakes collected from both depth and surface level during the six cold months, from November 2022 to April 2023. 

Concluding, the normal Raman spectroscopy experiments highlighted both the organic and inorganic differences between lakes for each month. 

### 3.3. SERS Spectra of Two Hypersaline Lake Waters over the Winter-Months Period

The abundant anions characteristic of salt water bodies, such as Cl^−^ and SO_4_^2−^, are strong aggregation agents for colloidal silver nanoparticles. Additionally, the presence and balance of nutrients were found to be crucial factors influencing the presence of the halophile microorganism community and thus providing specific molecular signatures. 

**Figure 4:**
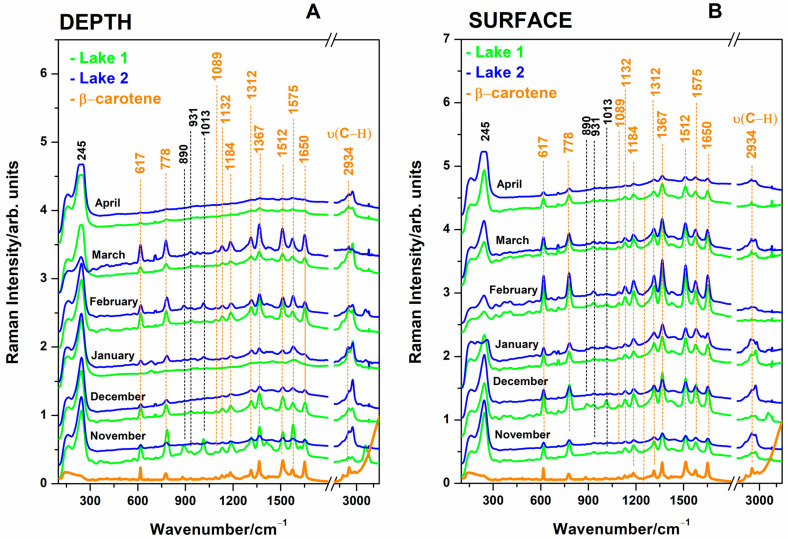
The SERS spectra collected from the salt waters of Lake 1 and Lake 2 from 1 m depth (**A**) and surface (**B**) waters over the six-month winter period, as indicated in each spectrum. Orange is shown for comparison in the SERS spectrum of pure β-carotene dissolved in ethanol solution, with SERS final concentration of 0.27 µM.

Surface and 1-meter-depth waters from the Cojocna hypersaline lakes were investigated in their raw form using SERS techniques. Analysis of the sampled waters using SERS revealed the presence of a very typical and reproducible signal attributable to β-carotene at sub-micromole concentrations [23,33], associated with the detection of cyanobacteria and comparatively displayed in Figure 4, for both depth and surface waters. This pattern was reproducible for both lake waters in terms of band positions and relative intensities, while absolute SERS intensity in identical experimental conditions was found to be different from one month to another. Each spectrum in Figure 4 is an average of three SERS-independent measurements of distinct subsamples of the respective month sampling. Spurious cases of subsampling, however, exhibited a weak SERS signal, or even no signal, in the range of 300–1800 cm^−1^, which was interpreted as excessive aggregation of nanoparticles in the presence of hypersaline water droplets. Since the pipette tips to probe microliter samples are the only variables in these experiments, we could suggest that a higher (microliter-range) amount of water produced rapid and excessive aggregation of AgNPs, which resulted in one huge SERS band at 245 in the overall SERS spectral range.

The observed SERS bands (Figure 4) at 1650, 1575, 1512, 1367, 1312, 1184, 1132, 1089, 778, and 617 cm^−1^ are assigned [23,33] to the chemisorbed β-carotene to the AgNPs surface, with the cyclohexene terminal rings of carotenoid structure attached or in close contact to the AgNPs surface. These bands are the characteristics of SERS bands of β-carotene at sub-micromole concentrations [23,33] and can be explained by admitting that AgNP clusters aggregated by salt ions are located in close contact with cyanobacteria rich in β-carotene. Since the concentration of this carotenoid in one live cyanobacteria is obviously small, the overall SERS events (referred to as the number of contacts between AgNP aggregates and individual cyanobacteria) contributed to the overall SERS signal. The higher number of cyanobacteria results in more intense SERS bands of β-carotene; thus, higher-intensity SERS bands mean a higher abundance of cyanobacteria. The fresh stock solution of pure β-carotene (Merck) in an ethanol solution was used to record the SERS reference spectrum at a final concentration of 0.27 µM. The SERS samples were made by adding 10 µL of β-carotene ethanol solution to 500 µL AgNPs colloidal solution and were used as comparative spectra.

The typical AgNPs aggregation in the presence of salt ions from waters exhibited a strong SERS band at 245 cm^−1^ attributable to Ag-Cl SERS adsorption. Thus, the signal intensity plots are proportional to the salt ion concentrations in lakes. It was expected that the SERS technique could potentially provide information on the organic molecules in waters, such as microalgae-released metabolites, cosmetic residues, pollutants, and other molecular species. However, the SERS typical pattern of adsorbed carotenoid was prevalent, and in a few cases, additional bands were observed along those of carotenoid, as highlighted with plotted dashed lines in Figure 4.

**Figure 5:**
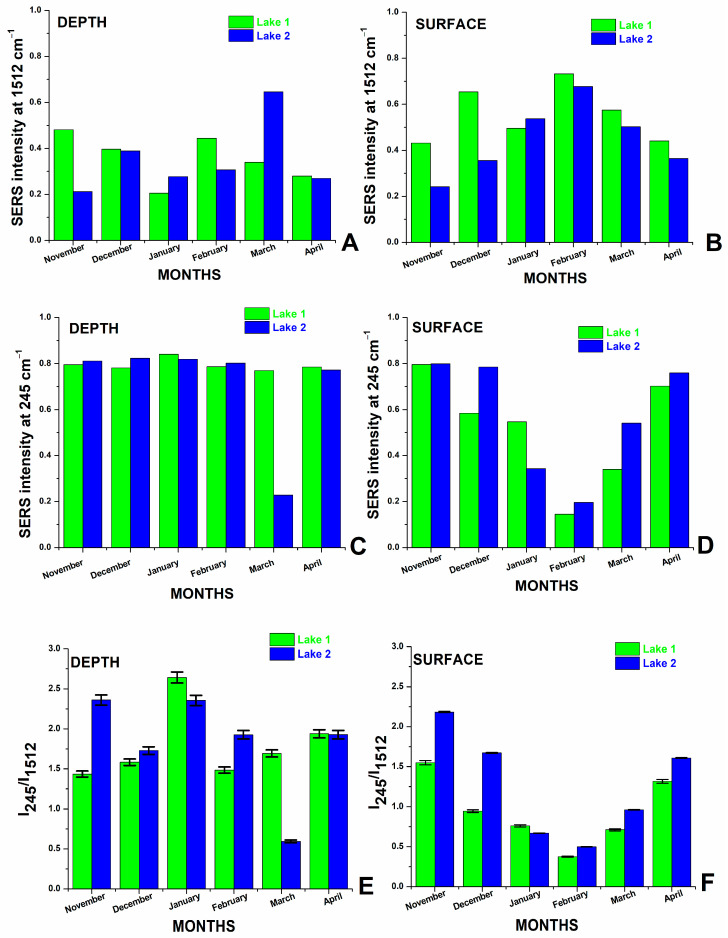
The plot of the SERS intensity band of carotenoids at 1512 cm^−1^ in samples from 1 m depth (**A**) and in surface waters (**B**) and the Ag-Cl band at 245 cm^−1^ for 1 m depth (**C**) and in surface waters (**D**) resulted from averaged data collected in each winter month from November to April, as indicated. The ratios calculated from the intensities of the bands in the SERS spectra of the waters show the variation of Cl-ions concentration compared with the organic carotenoids from cyanobacteria and reflect the monthly inorganic/organic balance of salt lakes in depth (**E**) and in surface waters (**F**). Error bars were inserted, representing a percentage of data values severally for each lake’s depths and surface waters.

The variation of the carotenoid SERS band intensity at 1512 cm^−1^ is plotted in Figure 5A,B, specific for each month of water investigation from 1 m depth and surface, respectively, while the Ag-Cl SERS band intensity at 245 cm^−1^ is displayed in Figure 5C,D for the case of 1 m depth waters (C) and surface waters (D). The Ag-Cl band at 245 cm^−1^ indicates stronger or lower aggregation, depending on the concentration of chloride ions. The ratios calculated from the intensities of the bands in the SERS spectra of the waters (Figure 5) show the variation of Cl^−^ concentration relative to the organic carotenoids (from cyanobacteria) and reflect the monthly inorganic/organic balance of salt lakes.

Cyanobacteria are one of the most widely distributed microorganisms across the world and have a remarkable ability to thrive in extreme environments [34,35,36]. Although the lake waters comprised a relatively large diversity of microorganisms, including various cyanobacteria species, the abundance of Dunaliella salina (green alga) was remarkable, as observed under light microscopy of water droplets. Dunaliella salina is also rich in β-carotene, and the interface between this alga and AgNP aggregated clusters could potentially contribute to the overall SERS pattern observed. Therefore, we examined several batches of SERS samples using transmission electron microscopy (TEM) analysis.

**Figure 6:**
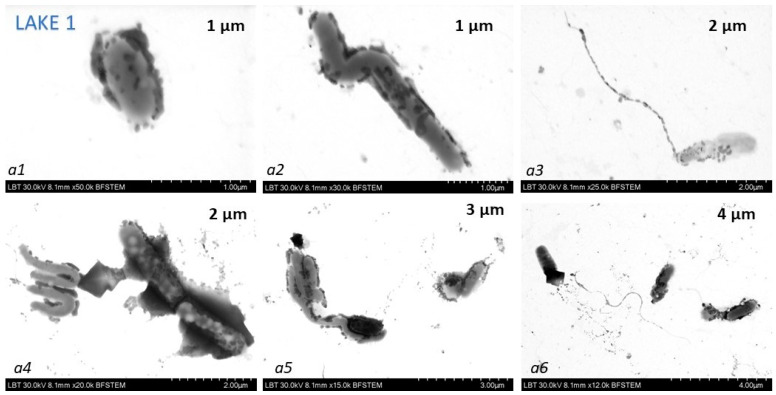

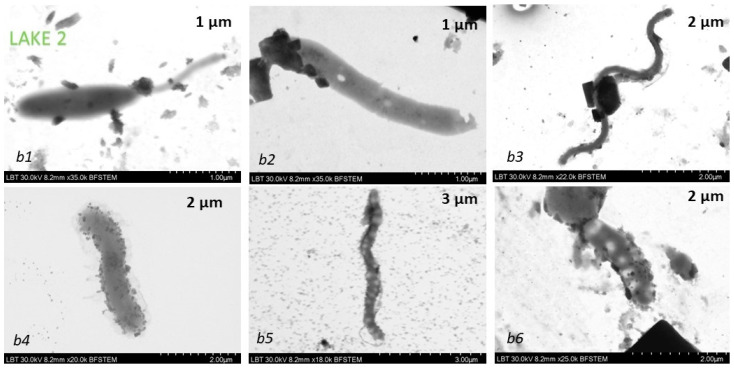
Transmission electron microscopy (TEM) images collected from droplets of SERS samples (**a1**–**a6** Lake 1 and **b1**–**b6** Lake 2) resulted from the salt waters—AgNPs interaction, suggesting many cyanobacteria species are in close contact with AgNPs aggregates. Scale bars are given in each bottom TEM image and range from 1 to 3 µm.

The TEM analysis provided information on the morphology and size of the AgNPs nanoparticles, their aggregates in the presence of ions from water, and interesting bacteria-AgNPs aggregate interfaces. A large diversity of bacteria-like shapes, e.g., spheroidal (Figure 6(a1,a6)), S-shaped (Figure 6(a2,b3,b5)) or rod-like (Figure 6(a3–a5,b1,b2,b4,b6)) microorganisms, appeared with attached AgNPs aggregates, and they are suggested as the main reason for the highly reproducible SERS spectral feature of the salt water-AgNPs complex in terms of band positions and relative intensities. TEM images show that the shapes and sizes varied considerably among the species found in hypersaline water bodies. Dunaliella salina (much larger than cyanobacteria) was not observed surrounded by or with attached AgNps clusters. Considering both the shape and dimensions of the observed microorganisms, as well as the characteristic SERS signal which resemble the reference SERS of β-carotene at sub micomole SERS concentrations [33] which we previously reported, we propose that the species of microorganisms responsible for the SERS signals in the salt waters are most likely cyanobacteria. This mechanism bears a SERS similarity to that previously reported in our Raman-SERS study of carbonate spring waters from several mountain springs from Swiss Alps [23].

## 4. Discussion

### 4.1. Statistical Correlation between Physicochemical Properties and Raman and SERS Signals of Lakes

The Pearson correlation coefficient was employed to facilitate a comprehensive investigation of the relationship between the Raman and SERS spectral data of two lake water samples and their respective physicochemical parameters (electrical conductivity, pH). This analysis aimed to elucidate how changes in relevant peak intensity within the Raman and SERS spectra correlate with fluctuations in pH and electrical conductivity over a six month timeline. 

We identified pairs that displayed significant correlations, whether positive or negative. A positive correlation indicates that two variables change simultaneously in the same direction, while a negative correlation suggests that they alternate in opposite directions. We considered correlation values above 0.5 as sufficiently significant for our analysis.

**Figure 7:**
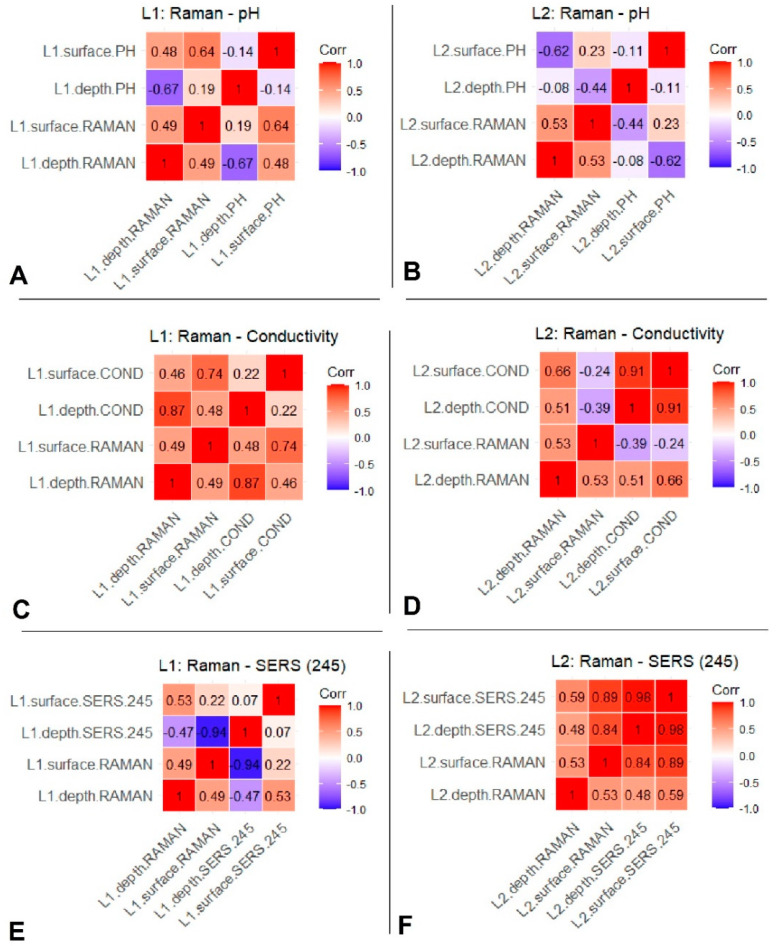
Representative correlation plots between datasets. (**A**) Correlation between Raman data and pH for Lake 1 and (**B**) Lake 2. (**C**) Correlation between Raman data and electrical conductivity for Lake 1 and (**D**) Lake 2. (**E**) Correlation between Raman and SERS data for Lake1 and (**F**) Lake 2.

Figure 7 illustrates the correlation coefficients between the Raman signal at 981 cm^−1^ of the sulfate and the measured pH and electrical conductivity, respectively, as well as the correlation between the Raman signal at 981 cm^−1^ and the SERS signal at 245 cm^−1^ of the Cl^−^ band. In Lake 1, a significant correlation is evident between the Raman signal at 981 cm^−1^ and the physicochemical parameters (Figure 7A,C). This suggests a strong positive linear relationship between Raman data and electrical conductivity values in both surface and deep water samples and the pH on the surface, but a negative correlation with the pH in depth. Conversely, in Lake 2, surface pH values negatively correlate with depth Raman data (Figure 7B), while a positive correlation is observed between depth Raman data on both depth and surface electrical conductivity (Figure 7D). Notably, no significant correlation is seen between the surface Raman data and pH, or electrical conductivity, in Lake 2.

A comprehensive examination of the association between Raman spectral data at 981 cm^−1^ and SERS data at 245 cm^−1^, shown in Figure 7E,F, reveals distinct correlation patterns. In Lake 1, there is a strong negative correlation between surface Raman data and SERS data at depth, while a good positive correlation is evident between depth Raman data and surface SERS. In Lake 2, a strong positive correlation is observed across all combinations when exploring the relationship between Raman and SERS spectral data.

**Figure 8:**
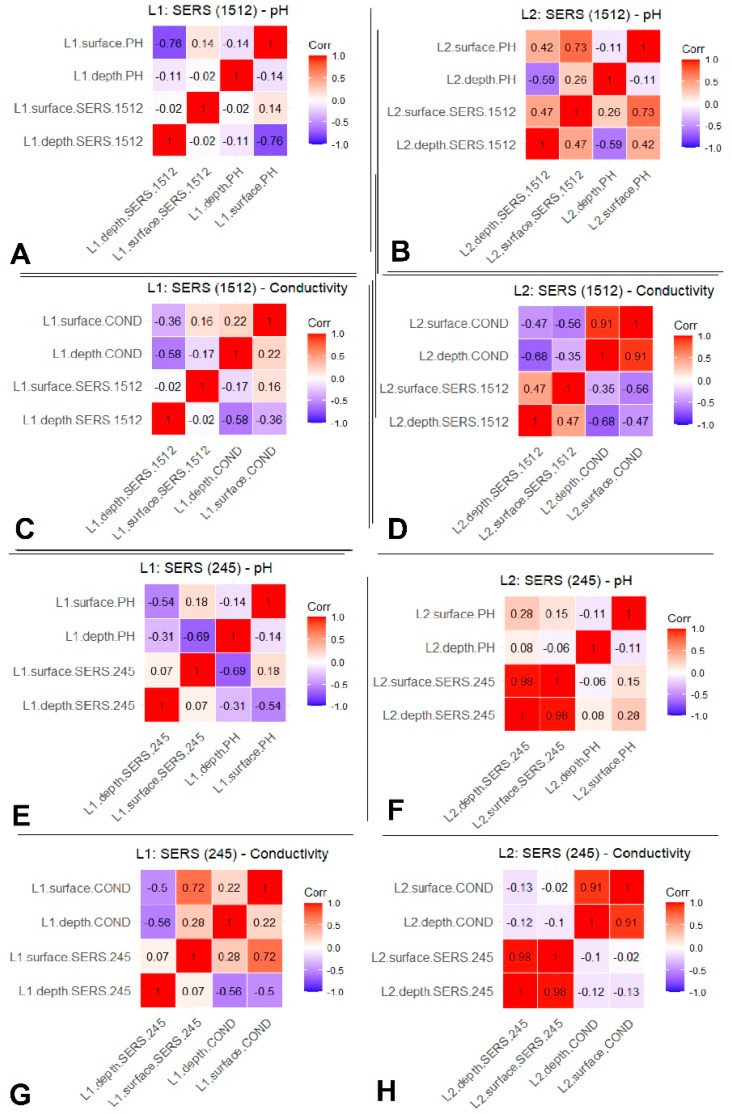
Representative correlation plots between datasets. Correlation between SERS spectral data at 1512 cm^−1^ and pH for (**A**) Lake 1 and (**B**) Lake 2; Correlation between SERS spectral data at 1512 cm^−1^ and electrical conductivity for (**C**) Lake 1 and (**D**) Lake 2; Correlation between SERS spectral data at 245 cm^−1^ and pH (**E**) Lake 1 and (**F**) Lake 2. Correlation between SERS spectral data at 245 cm^−1^ and electrical conductivity for (**G**) Lake 1 and (**H**) Lake 2.

Figure 8 displays the correlation coefficients between the SERS signals at 245 cm^−1^ of Cl^−^ band and at 1512 cm^−1^ of β-carotene band along with the measured pH and electrical conductivity.

The analyses revealed distinct patterns for the two lakes. Lake 1 showed a consistent positive correlation between the Raman data and physicochemical parameters, while Lake 2 exhibited a more complex relationship. In summary, the relationship between the Raman and SERS spectral data of the two lakes, along with their corresponding physicochemical parameters, exhibits a complex and distinctive pattern. The correlation analysis conducted for Lake 1 revealed a predictable relationship between the Raman spectral data at 981 cm^−1^, the physicochemical parameters, and the SERS spectral data at 245 cm^−1^. Similarly, significant correlations were noted for Lake 2 between the depth Raman spectral data and pH and electrical conductivity. These findings emphasize the complex and distinct nature of the relationship between spectral data and physicochemical parameters in the two lakes. The variations in the correlations underscore the unique dynamics governing the interplay between Raman and SERS data with pH and electrical conductivity, highlighting the significance of monitoring the physicochemical parameters of salt waters. These parameters act as essential indicators of the complex interactions between chemical constituents and environmental factors, emphasizing the need for tailored interpretations and considerations for each lake water system. The samples collected within a six-month timeline offered relevant water quality parameters that can be used in monitoring living (micro-) organisms and their ecosystems.

In Lake 1, a strong correlation is observed between the SERS signal at 1512 cm^−1^ from depths and the physicochemical parameters (Figure 8A,C). Notably, the depth SERS spectral data shows a negative correlation with surface pH data and depth electrical conductivity. Conversely, in Lake 2, a significant positive correlation is seen between the pertinent parameters and the SERS data at 1512 cm^−1^ (Figure 8B,D). Specifically, surface SERS data correlates strongly with surface pH, while depth SERS spectral data correlates inversely with depth pH values. Furthermore, a good negative correlation is noted between surface SERS data and surface electrical conductivity, as well as between depth SERS spectral data and depth electrical conductivity. These findings perfectly support the well-known physical situations when excessive AgNP aggregation hampers SERS detection of organic molecular species efficiency (high anions concentration, high conductivity, low SERS signal).

In Lake 1, a good correlation is also evident between the SERS spectral band at 245 cm^−1^ and the physicochemical parameters. However, no significant correlation is observed in the case of Lake 2. Further analysis in Lake 1 shows an inverse linear relationship between depth SERS spectral data and surface pH values and between surface SERS data and depth pH data (Figure 8E). Contrastingly, electrical conductivity data indicates a negative correlation between depth SERS spectral data and depth conductivity and a positive correlation between surface SERS spectral data and surface conductivity (Figure 8G). However, in the case of Lake 2, no notable correlation values are observed between the SERS data and the pH values (Figure 8F,H).

Based on the results from the correlation coefficient analyses (Figure 8), distinct patterns between the SERS signals are observed at 245 cm^−1^ for the Cl^−^ band and at 1512 cm^−1^ for the carotenoid band, along with measured pH and electrical conductivity in the two lakes. These parameters reflect the overall health of the aquatic ecosystem, and changes in parameters over the long term provide valuable data for studying trends and patterns in water quality, the presence of contaminants, or changes in the overall health of aquatic ecosystems.

The Pearson correlation coefficient analyses allowed for a comprehensive investigation of the spectral data of the two lakes and their respective physicochemical parameters, such as electrical conductivity and pH. The results underscore the complexity of interactions between nanostructures used as SERS amplifier substrates and the unique dynamics of the water samples investigated. It is important to note that, due to the small sample volume, the correlation coefficients calculated here are subjected to sensitivity fluctuations due to the background signal. Although optimized to record the best signal-to-background SERS data, external factors influencing higher background, such as living organism movement in a spotted droplet, cannot be avoided. Hence, other relationships between spectral data and physiochemical properties may be lost in the background or underestimated. By using statistical analysis, this drawback is reduced considerably, and in the following, we report on the unbiased, multivariate analysis of the spectral data by PCA.

### 4.2. Relative Band Intensities of Carotenoids Analysis with PCA of Two Lakes

PCA, or principal component analysis, is a widely used statistical technique for transforming a high-dimensional dataset into a lower-dimensional representation while retaining most of the relevant information. The goal of PCA is to find a set of orthogonal axes, called principal components (PCs), that capture the maximum variance in the data. These components are ranked in order of importance, with the first component explaining the most variance. By projecting the data onto a subset of PCs, PCA allows us to reduce the number of features or variables while minimizing the loss of information. This can serve as a mechanism to reveal patterns in multidimensional data that would otherwise remain hidden. 

For this purpose, we conducted PCA on a dataset comprising the SERS relative intensities of β-carotene on 13 bands (2972, 2930, 2882, 1651, 1574, 1512, 1365, 1311, 1184, 1133, 1085, 775, and 615 cm^−1^). The dataset was constructed in the following manner: the observations correspond to the relative band intensities on each individual band for both of the lakes respectively. The variables correspond to the depth profile at each month of sampling. 

**Figure 9:**
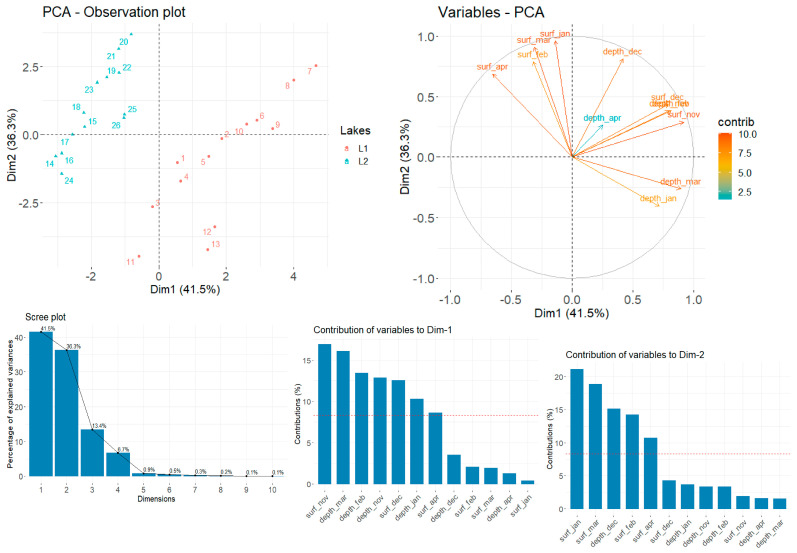
**TOP** (**Left**) PCA score plot shows the spectral discrimination between groups L1 and L2. (**Right**) Variable correlation plot on the first two dimensions. Variables corresponding to parallel vectors denote correlation, while perpendicularity is indicative of no variable correlation. **BOTTOM** (**Left**) Scree plot indicating the variance captured by each principal component. (**Right**) Contributions of variables to the first and second components, in decreasing order of strength.

The first two PCs managed to capture a total of 77.8% of the total explained variance (Figure 9). The distinct separation observed in the first principal component highlighted pronounced differences in the β-carotene band intensities between the two lakes, signifying potential variations in the composition or environmental factors influencing its distribution. These 13 band characteristics of SERS bands of β-carotene at sub-micromole concentrations [33] are specific to cyanobacteria. The higher number of cyanobacteria results in more intense SERS bands of β-carotene, so higher SERS bands mean a higher abundance of cyanobacteria.

Since the SERS signature profiles of β-carotene bands display distinct profiles among the two lakes (given the observations at various months and depth profiles throughout the year), we further investigated which sampling parameters contributed the most to the variance. The different month-depth sampling covariates that influence the first principal components the most are surface–November, depth–March, depth–February, depth–November, surface–December, and depth–January. Conversely, the second principal component has seen its highest contributions from surface–January, surface–March, depth–December, surface–February, and surface–April. We observed no dominant month or sampling profiles that strongly contribute to the L1-L2 band differentiation, since all months were well represented on the first two principal components.

### 4.3. PCA Analysis of Physics-Chemical Properties with SERS Signals of Lakes

**Figure 10:**
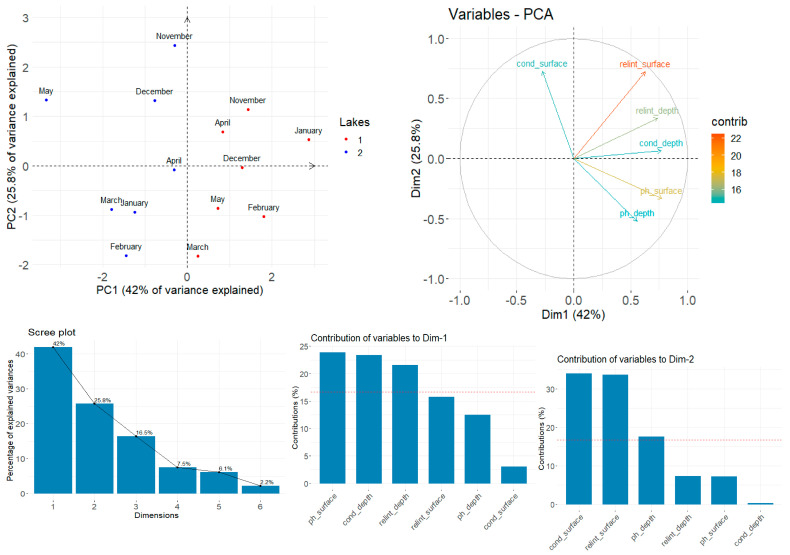
**TOP** (**Left**) PCA score plot shows the spectral discrimination between groups. (**Right**) PCA Variable plot showing association between variables and principal component. **BOTTOM** (**Left**) Scree plot indicating the percentage of variance captured by each principal component; (**Right**). Contributions to the variables to the first and second principal components.

We conducted a PCA analysis using the physicochemical parameters in order to assess whether they can also be used as discriminant markers for differentiating samples from L1 and L2. In this case, the first 2 PCs capture 67.8% of the total explained variance for this dataset (Figure 10). PC2 seems to roughly correspond with the variance stemming from the changing of seasons—samples from the spring months tend to be on the lower side of the y-axis, while samples from the winter months mostly localize on the upper side. 

Similar to our previous PCA, we investigated the association between variables and the first principal components. The conductivity, pH, and SERS relative band intensity all contributed equally to the differentiation, with no dominance associated with either deep or surface sampling origin. 

## 5. Conclusions

In this monitoring experiment, we employed nanotechnology-based techniques and instrumentation to explore the capability of Raman and SERS techniques to draw pertinent information regarding organic/inorganic content and dyanmics in two salt lakes used for balneotherapy. These measurements help to define the overall therapeutic properties concerning the dynamics of inorganic salt ions, contributing to a comprehensive understanding of the ecosystem’s health.

Here we present sensitive data relying on surface enhanced Raman scattering (SERS) signal monitoring of the molecular signature of salt lake water composition during the winter months (November 2022 to February 2023). AgNPs aggregation and SERS activity are strongly correlated with the presence of anions, primarily Cl^−^ and SO_4_^2−^ directly detected, but also others, and their concentration and synergic effect, while the presence and balance of nutrients are crucial for halophile microorganisms’ proliferation. Algal productivity is often correlated to levels of anions and their ratio, as well as with other water ions and other micronutrients. The anions characteristic of saltwater bodies are strong aggregation agents for colloidal silver nanoparticles generally used in surface-enhanced Raman scattering. To record reliable SERS spectra and get insight into the organic chemical content, the SERS technique required careful optimization of the sampling protocol regarding the volume ratio of added salt water and AgNPs. This dependence provides valuable insight into the aggregation dynamics of AgNPs in relation to the concentration of the anion and, consequently, the overall SERS signature. SERS data for water samples from two salt lakes are discussed and compared in a monitoring study aiming to trace organic changes associated with microbial metabolites.

We found significant spectral differences between water samples in two lakes, as well as significant chemical changes from one month to another. The SERS reproducibility in terms of band position and relative intensity was remarkable and not expected, and this feature was interpreted in terms of ubiquitous cyanobacteria presence in water droplets, which prompted the typical submicromole concentration SERS signal of β-carotene, an abundant carotenoid in cyanobacteria. As a matter of fact, we expected to record the SERS signature of aromatic substances resulting from microbial metabolites and decomposition, which are responsible for mud formation, as well as the possible inflow of aromatic residues either from the microbial community or from the presence of Anthropocene species at distinct levels within this timeline. A few exceptions from the regular SERS pattern of carotenoid were observed, such as the appearance of additional SERS bands at 2979 cm^−1^ in one or two cases, which, however, suggested that the multiplexed SERS pattern comes from multiple contributions, primarily cyanobacteria and possible other organic contributions. Overall, the Raman and SERS data on the salt lake’s water, perfectly compatible with in situ monitoring initiatives, clearly highlighted and differentiated two adjacent salt water bodies in terms of both organic and inorganic composition and dynamics, although both water bodies benefit from identical climate conditions and anthropogenic influence, specifically for balneary resort exploitation. Organic/inorganic balance is a crucial characteristic for decision-making regarding any forthcoming therapeutic effect or potential investigation of the hypersaline water bathing approach.

## Data Availability

Data are contained within the article.

## Supplementary Materials

The following supporting information can be downloaded at: https://www.mdpi.com/article/10.3390/bios14010019/s1. Figure S1. UV-vis spectra of the silver colloidal solution; Figure S2. Transmission electron microscopy (TEM) image of silver nanoparticles; Figure S3. The Temperature (°C) of the lakes during winter months; Figure S4. The measured Absorbance values of chlorophylls (668 nm) (bottom) and carotenoids (470 nm) (upper) from electronic absorption spectra of raw water samples collected each month of the studied period; Table S1. The absorbance of chlorophyll and carotene from water samples each month.

## References

[1] Baricz A., Levei E.A., Șenilă M., Pînzaru S.C., Aluaş M., Vulpoi A., Filip C., Tripon C., Dădârlat D., Buda D.M. (2021). Comprehensive Mineralogical and Physicochemical Characterization of Recent Sapropels from Romanian Saline Lakes for Potential Use in Pelotherapy. Sci. Rep..

[2] Ionescu E.V., Suta M., Surdu O., Oprea C., Stoicescu R.M., Taralunga G., Lilios G. (2014). Clinical and Biological Effects Induced by Sapropelic Mud from the Lake Techirghiol in Patients with Osteoarthritis. J. Environ. Prot. Ecol..

[3] Katz U., Shoenfeld Y., Zakin V., Sherer Y., Sukenik S. (2012). Scientific Evidence of the Therapeutic Effects of Dead Sea Treatments: A Systematic Review. Semin. Arthritis Rheum..

[4] Pinzaru T. (1971). Complexul Lacustru de La Cojocna (article in Romanian). Stud.Geogr..

[5] Şerban G., Alexe M., Touchart L. (2005). Morphological Evolution and Salinity of Cojocna Lakes (Transylvanian Plain, Romania). Bull. d’Association Geogr. Fr..

[6] Krézsek C., Filipescu S. (2005). Middle to Late Miocene Sequence Stratigraphy of the Transylvanian Basin (Romania). Tectonophysics.

[7] Somogyi B., Vörös L., Pálffy K., Székely G., Bartha C., Keresztes Z.G. (2014). Picophytoplankton Predominance in Hypersaline Lakes (Transylvanian Basin, Romania). Extremophiles.

[8] Alexe M., Șerban G., Andreea B., Andrei A.-Ș., Adorján C., Battes K.P., Cîmpean M., Momeu L., Muntean V., Porav S.A. (2018). Limnology and Plankton Diversity of Salt Lakes from Transylvanian Basin (Romania): A Review. J. limnol..

[9] Czellecz B., Gabor I., Ravasz L., Schiopu G., Szopos N. (2003). Saline Water Resurces in Cluj-Napoca Surroundings.

[10] Gheorghievici L., Gheorghievici G., Tănase I. (2015). The Phytoplankton Composition Features of Five Romanian Pelogenous Ecosystems. Environ. Eng. Manag. J..

[11] Keresztes Z.G., Felföldi T., Somogyi B., Székely G., Dragoş N., Márialigeti K., Bartha C., Vörös L. (2012). First Record of Picophytoplankton Diversity in Central European Hypersaline Lakes. Extremophiles.

[12] Brezestean I., Nekvapil F., Cinta Pinzaru S. (2018). Analysis of Hypersaline Waters from Cojocna Balneary Resorts (Romania) Using Raman Spectroscoy Techniques. https://www.researchgate.net/publication/323854857_Analysis_of_hypersaline_waters_from_Cojocna_balneary_resorts_Romania_using_Raman_spectroscopy_techniques.

[13] Corral P., Amoozegar M.A., Ventosa A. (2020). Halophiles and Their Biomolecules: Recent Advances and Future Applications in Biomedicine. Mar. Drugs.

[14] Oren A., Rodriguez-Valera F. (2001). The Contribution of Halophilic Bacteria to the Red Coloration of Saltern Crystallizer Ponds. FEMS Microbiol. Ecol..

[15] Marucci G., Beeby A., Parker A.W., Nicholson C.E. (2018). Raman Spectroscopic Library of Medieval Pigments Collected with Five Different Wavelengths for Investigation of Illuminated Manuscripts. Anal. Methods.

[16] Ishihara J.I., Takahashi H. (2023). Raman Spectral Analysis of Microbial Pigment Compositions in Vegetative Cells and Heterocysts of Multicellular Cyanobacterium. Biochem. Biophys. Rep..

[17] Tewes T.J., Kerst M., Platte F., Bockmühl D.P. (2022). Raman Microscopic Identification of Microorganisms on Metal Surfaces via Support Vector Machines. Microorganisms.

[18] Cui D., Kong L., Wang Y., Zhu Y., Zhang C. (2022). In Situ Identification of Environmental Microorganisms with Raman Spectroscopy. Environ. Sci. Ecotechnol..

[19] Oliva-Teles L., Pinto R., Vilarinho R., Carvalho A.P., Moreira J.A., Guimarães L. (2022). Environmental Diagnosis with Raman Spectroscopy Applied to Diatoms. Biosens. Bioelectron..

[20] Li Z., Wang J., Li D. (2016). Applications of Raman Spectroscopy in Detection of Water Quality. Appl. Spectrosc. Rev..

[21] Jehlička J., Edwards H.G., Oren A. (2014). Raman Spectroscopy of Microbial Pigments. Appl. Environ. Microbiol..

[22] Wieser W., Assaf A.A., Gouic B.L., Dechandol E., Herve L., Louineau T., Dib O.H., Gonçalves O., Titica M., Couzinet-mossion A. (2023). Development and Application of an Automated Raman Sensor for Bioprocess Monitoring: From the Laboratory to an Algae Production Platform. Sensors.

[23] Cinta Pinzaru S., Ardeleanu M., Brezestean I., Nekvapil F., Venter M.M. (2019). Biogeochemical Specificity of Adjacent Natural Carbonated Spring Waters from Swiss Alps Promptly Revealed by SERS and Raman Technology. Anal. Methods.

[24] Müller Molnár C., Cintă Pînzaru S., Chis V., Feher I., Glamuzina B. (2023). SERS of Cylindrospermopsin Cyanotoxin: Prospects for Quantitative Analysis in Solution and in Fish Tissue. Spectrochim. Acta Part A Mol. Biomol. Spectrosc..

[25] Pinzaru S.C., Müller C., Ujević I., Venter M.M., Chis V., Glamuzina B. (2018). Lipophilic Marine Biotoxins SERS Sensing in Solutions and in Mussel Tissue. Talanta.

[26] Müller C., Glamuzina B., Pozniak I., Weber K., Cialla D., Popp J., Cîntǎ Pînzaru S. (2014). Amnesic Shellfish Poisoning Biotoxin Detection in Seawater Using Pure or Amino-Functionalized Ag Nanoparticles and SERS. Talanta.

[27] Lee P.C., Meisel D. (1982). Adsorption and Surface-Enhanced Raman of Dyes on Silver and Gold Sols. J. Phys. Chem..

[28] Jiang Y., Wang X., Zhao G., Shi Y., Thuy N.T.D., Yang H. (2022). SERS Determination of Trace Phosphate in Aquaculture Water Based on a Rhodamine 6G Molecular Probe Association Reaction. Biosensors.

[29] Wei T., Simko V., Levy M., Xie Y., Jin Y., Zemla J. (2017). Package ‘corrplot’. Statistician.

[30] Lê S., Josse J., Husson F. (2008). FactoMineR: An R Package for Multivariate Analysis. J. Stat. Softw..

[31] Kassambara A., Mundt F. (2017). Package ‘Factoextra’. Extract and Visualize the Results of Multivariate Data Analyses.

[32] Lichtenthaler H.K., Buschmann C. (2001). Chlorophylls and Carotenoids: Measurement and Characterization by UV—VIS Spectroscopy. Curr. Protoc. Food Anal. Chem..

[33] Cintə Pinzaru S., Müller C., Tomšic S., Venter M.M., Cozar B.I., Glamuzina B. (2015). New SERS Feature of β-Carotene: Consequences for Quantitative SERS Analysis. J. Raman Spectrosc..

[34] Bell S.E.J., Sirimuthu N.M.S. (2005). Surface-Enhanced Raman Spectroscopy as a Probe of Competitive Binding by Anions to Citrate-Reduced Silver Colloids. J. Phys. Chem. A.

[35] Cinta S., Pinzaru, Csilla M.M., Ioana B., Glamuzina B. (2016). Cyanobacteria Detection and Raman Spectroscopy Characterization with a Highly Sensitive, High Resolution Fiber Optic Portable Raman System. Stud. Univ. Babes-Bolyai Phys..

[36] Antón J., Oren A., Benlloch S., Rodríguez-Valera F., Amann R., Rosselló-Mora R. (2002). Salinibacter Ruber Gen. Nov., Sp. Nov., a Novel, Extremely Halophilic Member of the Bacteria from Saltern Crystallizer Ponds. Int. J. Syst. Evol. Microbiol..

