# Supplementary material for: Pilot SERS Monitoring Study of Two Natural Hypersaline Lake Waters from a Balneary Resort during Winter-Months Period
Source: Biosensors (Basel). 2023 Dec 29;14(1):19. doi: 10.3390/bios14010019 (PMC10813592; doi:10.3390/bios14010019)
Supplement: Supplementary file 1 [file biosensors-14-00019-s001.zip › biosensors-2726737-supplementary.pdf]

biosensors

Supplementary Materials

Pilot SERS Monitoring Study of Two Natural Hypersaline Lake Waters from a Balneary Resort during Winter-Months Period

Csilla Molnár [1,2,*], Teodora Diana Drigla [2], Lucian Barbu-Tudoran [3], Ilirjana Bajama [2], Victor Curean [4] and Simona Cîntă Pînzaru [2,5,*]

1 National Institute for Research and Development of Isotopic and Molecular Technologies, 67-103 Donath, 400293 Cluj-Napoca, Romania
2 Biomolecular Physics Department, Babeş-Bolyai University, Kogălniceanu 1, 400084 Cluj Napoca, Romania; teodoradrigla@yahoo.com (T.D.D.); ilirjana.bajama@ubbcluj.ro (I.B.)
3 Electron Microscopy Centre, Babeș-Bolyai University, Clinicilor 5-7, 400006 Cluj-Napoca, Romania; lucian.barbu@itim-cj.ro
4 Faculty of Pharmacy, "Iuliu Hatieganu" University of Medicine and Pharmacy, Victor Babes 8, 400347 Cluj-Napoca, Romania; curean.victor@elearn.umfcluj.ro
5 Institute for Research, Development and Innovation in Applied Natural Sciences, Babes-Bolyai University, Fantanele 30, 400327 Cluj-Napoca, Romania
* Correspondence: csilla.molnar@itim-cj.ro (C.M.); simona.pinzaru@ubbcluj.ro (S.C.P.)

The UV-VIS spectrum of colloidal nanoparticles

The electronic absorption spectrum of silver colloidal nanoparticles exhibited a band centered at 417 nm. The optical absorbance of the obtained silver nanoparticles was measured using a UV-Vis Shimadzu 1900 spectrophotometer.

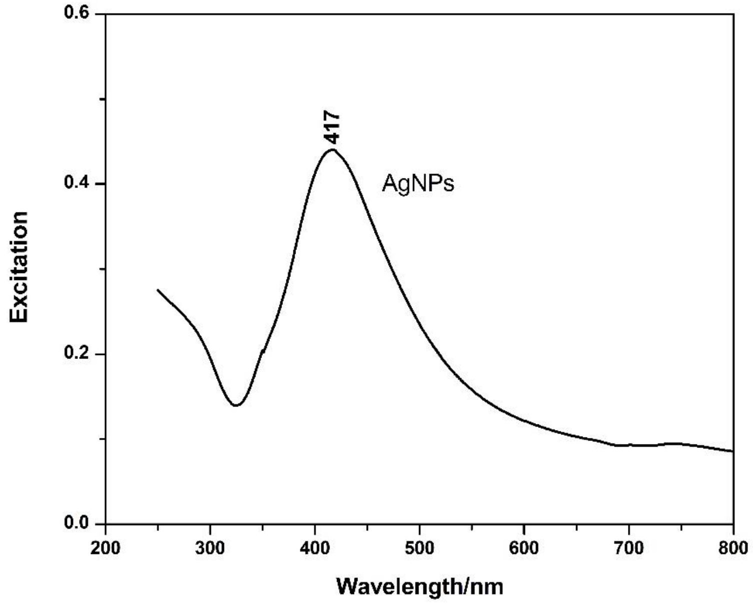

Figure S1. UV-vis spectra of the silver colloidal solution.

The size and morphology of the silver nanoparticles was characterized by transmission electron microscopy in conjunction with the energy dispersive spectroscopy (TEM-EDX) (Fig. S2), using a Hitachi SU8230 cold field emission scanning transmission electron microscope, Hitachi, Japan.

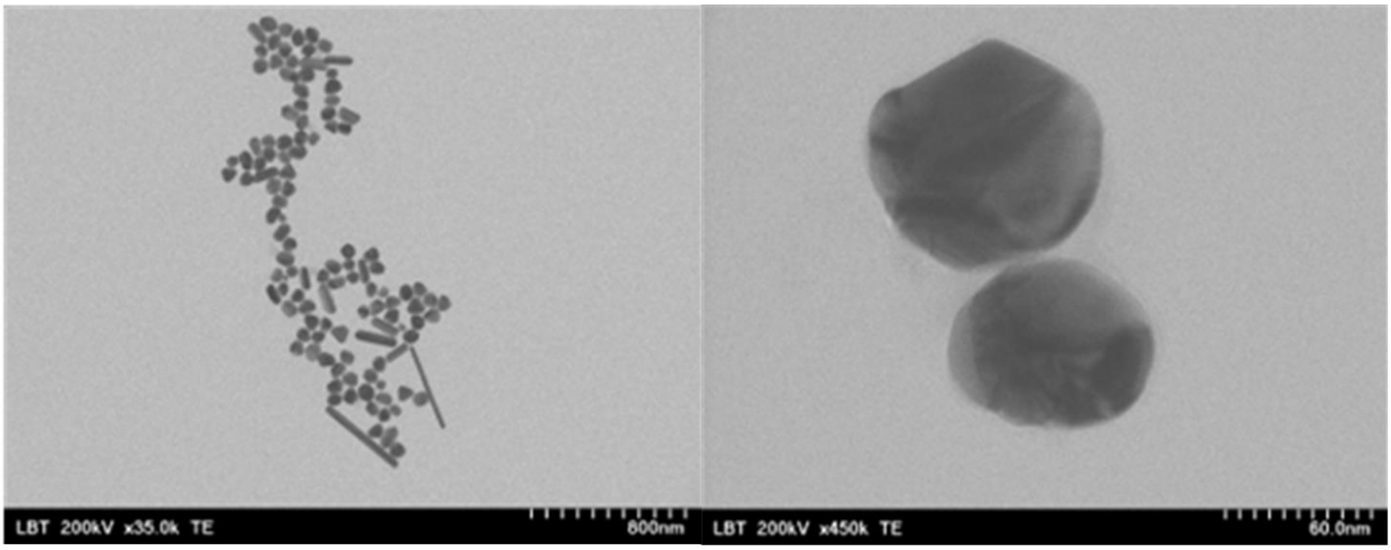

Figure S2. Transmission electron microscopy (TEM) image of silver nanoparticles

The TEM image (Figure S2) revealed that the AgNPs were spherically in shape, uniformly distributed.

This stock of AgNPs was used for all the SERS measurements

The water temperature (˚C) of the lakes during the winter month

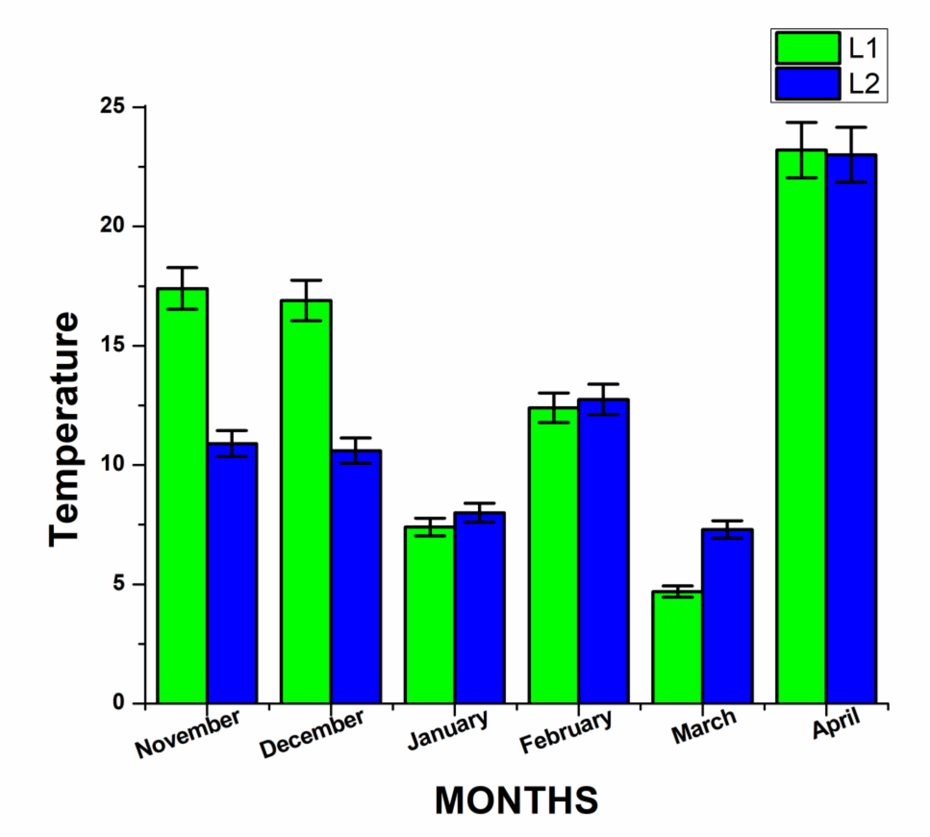

Figure S3. The Temperature (˚C) of the lakes during winter months

The UV-VIS spectrum of water samples

In the context of water analysis, UV-VIS spectroscopy was employed to detect and quantify the carotenoid and chlorophyll content. These exhibit characteristic absorption spectra in the UV-Vis region. Carotenoids absorb light in the visible range, particularly in the blue and green regions, resulting in their distinctive color. Chlorophyll, on the other hand, absorbs strongly in the blue and red regions of the spectrum, with minimal absorption in the green region, which gives algae their green appearance [32].

The absorbance of water samples from two lakes was measured from the UV-Vis spectra using incident light of wavelength equal to 470 nanometers for carotene and 668 nanometers for chlorophyll, according to the literature [33]. The results are shown in table S1 and Figure S3.

Table S1. The absorbance of chlorophyll and carotene from water samples each month

Month	Lake	Absorbance value	
		Carotene	Chlorophyll
November	L1	0.0090	0.0020
	L2	0.0073	0.0043
December	L1	0.0547	0.0333
	L2	0.0005	0.0020
January	L1	0.0723	0.0677
	L2	0.0100	0.0060
February	L1	0.0683	0.0523
	L2	0.0083	0.0063
March	L1	0.0303	0.0233
	L2	0.0443	0.0210
April	L1	0.0350	0.0110
	L2	0.0170	0.0050
May	L1	0.0153	0.0147
	L2	0.0100	0.0133

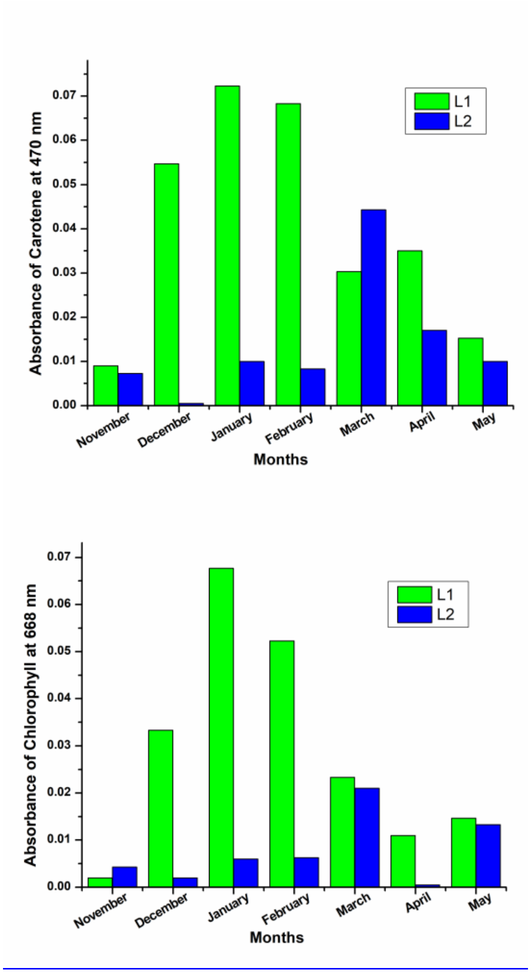

Figure S4. The measured Absorbance values of chlorophylls (668 nm) (bottom) and carotenoids (470 nm) (upper) from electronic absorption spectra of raw water samples collected each month of the studied period
